# Determinants of natal dispersal distances in North American birds

**DOI:** 10.1002/ece3.9789

**Published:** 2023-02-08

**Authors:** Jonathan J. Chu, Santiago Claramunt

**Affiliations:** ^1^ Department of Ecology and Evolutionary Biology University of Toronto Toronto Ontario Canada; ^2^ Department of Natural History Royal Ontario Museum Toronto Ontario Canada

**Keywords:** birds, ecomorphology, emigration, flight efficiency, mobility, wing morphology

## Abstract

Natal dispersal—the movement from birth site to first breeding site—determines demographic and population genetic dynamics and has important consequences for ecological and evolutionary processes. Recent work suggested that one of the main factors determining natal dispersal distances is the cost of locomotion. We evaluated this hypothesis using band recovery data to estimate natal dispersal distances for 50 North American bird species. We then analyzed the relationships between dispersal distances and a suite of morphological and ecological predictors, including proxies for the cost of locomotion (flight efficiency), using phylogenetic regression models. We found that flight efficiency, population size, and habitat influence natal dispersal distances. We discuss how the effects of population size and habitat can also be related to mobility and locomotion. Our findings are consistent with a predominant effect of adaptations for mobility on dispersal distances.

## Graphical abstract

Dispersal is fundamental to many processes in ecology and evolution. We evaluated the hypothesis that the cost of movement is most influential in dispersal process in North American birds. We found that flight efficiency, population size, and habitat influence natal dispersal distance, although the individual effects are difficult to disentangle.

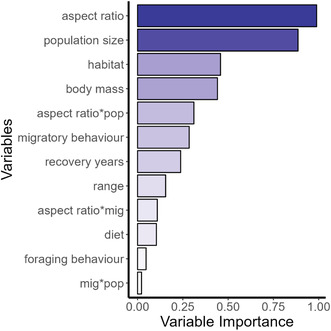

## 1 INTRODUCTION

Dispersal is an important aspect of ecology and evolution that influences many processes such as population spatial dynamics, species distributions, gene flow, and speciation (Bohonak, 1999; Bowler & Benton, 2005; Claramunt et al., 2012; Gaston, 2003; Hanski, 1998; Greenwood, 1980; Pigot & Tobias, 2015). Despite its importance, the factors that influence dispersal distances are poorly understood. Most theories have framed dispersal as an adaptive strategy for individuals facing competition, inbreeding, or variation in resource abundance (Bowler & Benton, 2005; Clobert et al., 2009; Matthysen, 2005; McCaslin et al., 2020; Moore & Aki, 1984; Nelson‐Flower et al., 2012). In those cases, dispersal distances are predicted to be longer in species that live in dense populations with high rates of inbreeding. But empirical evidence supporting these predictions has been ambiguous (Duputié & Massol, 2013; Matthysen, 2012; Paradis et al., 1998; Ronce, 2007). On the other hand, dispersal distances might not reflect an adaptive strategy but instead, a by‐product of movements intended for other activities such as foraging and commuting (Burgess et al., 2016; Claramunt, 2021; Matthysen, 2012).

The lack of empirical evidence supporting theoretical predictions can be attributed in part to the difficulties in collecting dispersal distance data. At the most basic level, measuring dispersal distance requires an organism to be marked and later recaptured. But recapture rates are typically low and local studies can miss the recapture of individuals that have dispersed out of the study area, leading to an underestimation of dispersal distances (Koenig et al., 1996; Tittler et al., 2009). This, plus varying sampling methods and geographical scales have made the comparative analysis of dispersal distances difficult. Paradis et al. (1998) used the countrywide and long‐term bird banding data from the British Trust for Ornithology to estimate dispersal distances for 75 British bird species allowing for direct comparisons and comparative analysis (Claramunt, 2021; Dawideit et al., 2009; Garrard et al., 2012). This dataset revealed that, contrary to theoretical expectations, body size and life history characteristics were largely unrelated to dispersal distances (Claramunt, 2021; Paradis et al., 1998). Also, at odds with theoretical expectations based on competition and inbreeding avoidance, population size was negatively correlated with dispersal distances (Claramunt, 2021; Paradis et al., 1998). Migratory species tend to disperse further (Dawideit et al., 2009; Paradis et al., 1998) but this may be due to their high flight efficiency rather than an effect of migratory movements per se (Claramunt, 2021). Evidence for the influence of habitat or diet on dispersal distances is still ambiguous (Claramunt, 2021; Paradis et al., 1998). Overall, the factors emerging as most influential were proxies of long‐distance flight efficiency, suggesting a predominant role for the cost of movement on dispersal patterns (Claramunt, 2021). However, all these findings are based on an analysis of British birds. It is not known if avifaunas in other parts of the world show similar patterns, but some evidence points to this being the case (Weeks et al., 2022).

In this study, we set out to fill this gap by analyzing factors that influence natal dispersal distances in North American birds. We estimated natal dispersal distances by using data from the North American bird banding program following the approach of Paradis et al. (1998). We conducted comparative analyses to assess the influence of flight efficiency and behavioral and ecological traits that have been theorized to influence natal dispersal distances (Garrard et al., 2012; Paradis et al., 1998; Ronce, 2007; Sutherland et al., 2013). By analyzing these results and comparing them with those from British birds, we discuss the underlying factors that seem to be controlling variation in natal dispersal distances in birds.

## 2 METHODS

### 2.1 Estimation of natal dispersal distances

Dispersal movements can be split into two categories, natal dispersal, defined as the movement of an animal from birth site to first breeding site, and breeding dispersal, defined as the movement of a breeding adult between breeding sites in subsequent years. This study focuses on variation in natal dispersal distance as it is usually the longest dispersal event among birds, and thus the most influential to gene flow, population dynamics, and metacommunity connectivity (Greenwood & Harvey, 1982; Lester et al., 2007; Paradis et al., 1998). We used records from the North American bird banding program, conducted jointly by the Canadian Wildlife Service and the United States Geological Survey (Buckley et al., 1998), to estimate natal dispersal distances following the methods outlined by Paradis et al. (1998).

The records we used spanned bandings and recoveries from 1920 to 2019. We obtained all recovery records of birds banded as nestlings or fledglings to ensure that the banding site reflects the natal site. The distance between this point and the subsequent recovery location was taken as an estimate of the individual's natal dispersal distance, given the additional following conditions. Only birds banded and recaptured during the species' breeding season and within the species' breeding range were considered in order to avoid the incorporation of migratory movements. Information on breeding seasons was obtained from species accounts collated in Birds of the World (Billerman et al., 2020). Given the variation in breeding seasons within species, we took the earliest month and latest month reported to be the bounds. To determine breeding range limits, the most extreme points on shapefiles of the species' distribution were set as latitude and longitude filters (BirdLife International, 2019). Only birds recovered as mature adults were considered to ensure that the recapture location represented a potential breeding site. We discarded records with location uncertainty greater than 1′ block. To limit spatial biases in the location of recoveries due to human activities, only birds found dead were used, thus excluding recoveries at banding stations or hunting sites. The resultant banding and recovery data were plotted on maps of North America to inspect if filters were successful in limiting spurious effects and recoveries along migratory routes.

Distances between banding and recovery sites, in kilometers, were measured using the R package “geosphere” using the “Vincenty” (ellipsoid) method (Hijmans, 2019). This method measures the shortest distance between two points on an ellipsoid approximating Earth's actual shape and should be the most accurate estimation for the shortest distance between two geographic locations (Vincenty, 1975). We then calculated the geometric mean of dispersal distances for each species. Because standard errors in natal dispersal distance estimates increased exponentially for species with fewer than five recoveries, only species with a sample size greater than four were retained for further analyses.

This method uses the distance between the fledgling site and any recovery site after maturity as opposed to only individuals recovered in their first breeding site, which may be difficult to infer from banding data alone. This may result in some breeding dispersal events being added to the estimated natal dispersal distances. However, because breeding dispersal is usually less frequent and shorter than natal dispersal, we expect this bias to be minor. In any case, to account for this potential bias, we calculated the mean number of years between banding and recovery for each species and included it as a covariate in our analyses.

### 2.2 Predictors

We used the aspect ratio of the wings as a proxy for flight efficiency. The aspect ratio is the morphological characteristic of the wing that is most influential in determining long‐distance flight efficiency (Pennycuick, 2008) and is calculated as:

$$\frac{B^{2}}{A_{\mathrm{tot}}}$$

where *B* is the wingspan and *A*_tot_ is the total wing area (the area of both wings plus the area of the body between the wings) estimated as:

$$A_{\mathrm{tot}}=2A_{w}+C_{r}∙\left(B-2E\right)$$

in which *A*_*w*_ is the area of a single wing, *C*_*r*_ is the root chord, the width of the wing at its proximal border, and *E* is the wing extent, measured as the distance between the root chord (perpendicular) and the most distant feather tip (see Pennycuick [2008] and Claramunt and Wright [2017] for illustrations and further details).

Estimates of single‐wing areas were obtained from spread wings photographed at the Royal Ontario Museum and from the digital collection of spread‐wing images of the Slater Museum of Natural History (https://digitalcollections.pugetsound.edu/digital/collection/slaterwing). Wingspan data were taken from museum specimen records in VertNet (vertnet.org). Additional data on wing area and wingspan were obtained from the Wings Database (Pennycuick, 2008) included in the Flight 1.25 software (https://booksite.elsevier.com/9780123742995/?ISBN=9780123742995). These data were used to estimate species' averages and do not correspond to the individual birds used to estimate natal dispersal distances.

We used ImageJ v.1.52 (Schneider et al., 2012) to process and measure wing images. We set the scale using the scale bars included in the images and transformed the image into a binary image using the thresholding tool. We then measured the area of the wing ($A_{w}$) using the Analyze Particles tool. The root chord ($C_{r}$) and the wing extent (E) were measured using the straight‐line tool.

We also estimated and tested two alternative flight efficiency proxies—the hand‐wing index and the lift‐to‐drag ratio—for which we describe methods and present results in Appendix A.

Two geographical predictors—migratory distance and breeding range area—were obtained from geographic range shapefiles (BirdLife International, 2019). Shapefiles were analyzed using QGIS v.3.14 (QGIS Development Team, 2020). We used the distance between the centroids of the breeding and the wintering range as an estimate of the species migration distance (using the “Vincenty” method). We estimated the breeding range area (in squared kilometers) using the $area command in QGIS.

Ecological predictors were habitat, diet, foraging behavior, and population size. Habitat, diet, and foraging behavior information were obtained from species accounts collated in Birds of the World (Billerman et al., 2020). Habitat was divided into four categories: woodlands, open habitats, wetlands, and coasts. Species that inhabit aquatic ecosystems were split between wetlands and coasts, depending on whether their habitat consists of interior lakes, wetlands, and rivers (wetlands) or beaches, marine coastlines, and open oceans (coasts). Woodlands include forests and woodlands, and open habitats include grasslands, deserts, and steppes. Diet was divided into four categories: herbivores, species that primarily feed on plants; carnivores, species that primarily feed on vertebrates; insectivores, species that primarily feed on insects and other invertebrates; and omnivores, species that feed on both plants and animals. Foraging behavior was categorized into five groups, effectively an ordinal variable that corresponded to a continuum of the amount of flight required to forage. The first group, surface foraging, includes species that primarily forage while walking, wading, or floating so flight is not needed for prey detection and capture (e.g., Mourning Dove, *Zenaida macroura*, Otis et al., 2020). The second level, tree foraging, refers to species that forage in elevated vegetation like arboreal insect gleaners and tree climbers, that need to fly from branch to branch or from tree to tree during foraging (e.g., Red‐cockaded Woodpecker, *Dryobates borealis*, Jackson, 2020). The third level, sallying, refers to species that take off from perches to pursue prey after which they return to a perch (e.g., Least Flycatcher, *Empidonax minimus*, Tarof & Briskie, 2020). The fourth level, aerial search, refers to species that search for prey in flight but dive down to capture prey on the ground or in the water (e.g., Red‐tailed Hawk, *Buteo jamaicensis*, Preston & Beane, 2020). The fifth level, aerial capture, refers to species that search for, capture, and ingest prey on the wing (e.g., Purple Martin, *Progne subis*, Brown et al., 2021). Population sizes were taken from Rosenberg et al. (2019) with data originally published by Partners in Flight (Stanton et al., 2019). Population sizes represent the breeding population size across the species' entire range in the United States and Canada estimated from the North American Breeding Bird Survey (Sauer et al., 2017). The population size of the American Oystercatcher, *Haematopus palliatus*, was obtained from Andres et al. (2012).

### 2.3 Statistical analysis

Relationships between predictors and natal dispersal distance were assessed using phylogenetic generalized least squares models (PGLS) to account for phylogenetic non‐independence among species (Freckleton et al., 2002). PGLS models were fit by maximum likelihood with the *pgls* function in the R package “caper” (Orme et al., 2018). Phylogenetic non‐independence (phylogenetic inertia or signal) is incorporated into the error term of PGLS models by specifying an error–covariance matrix representing the shared phylogenetic history between species pairs (shared branches in time units), transformed by a parameter (*λ*) that moderates the intensity of phylogenetic inertia in relation to the strict Brownian motion expectation (Freckleton et al., 2002; Pagel, 1999). The phylogenetic tree used was a maximum clade credibility tree computed using TreeAnnotator (Bouckaert et al., 2014) from a sample of 1000 phylogenetic trees of the study species obtained from Birdtree.org (Jetz et al., 2012) using the Hackett et al. (2008) backbone topology. We also used Pagel's *λ* model (Pagel, 1999) to assess levels of phylogenetic signal in dispersal distances per se using function phylosig in the phytools package (Revell, 2012). Continuous variables were transformed by the natural logarithm to increase homoscedasticity, in the case of natal dispersal distances (Faraway, 2005), and to improve model likelihoods and the distribution of residuals for flight efficiency, migration distance, mean number of years between banding and recovery, and population size predictors.

We explored all predictors individually in single‐predictor models and then constructed multi‐predictor models with main effects and second‐order effects (interactions) between continuous predictors and binary predictors. We did not consider models with more than five variables and greater than second‐order effects. Models were assessed using model selection and multi‐model inferences techniques using the Akaike information criterion (AICc) and relative model probabilities (Burnham & Anderson, 2002). Predictors were standardized by subtracting the mean and dividing by 1 standard deviation before analyses. Model fit and proportion of variance explained by the models were assessed by calculating coefficients of determination:

$$R^{2}=1-\frac{{\mathrm{RSS}}_{\text{model}}}{{\mathrm{SS}}_{\text{null}}}$$

in which RSS_model_ is the residual sum of squares of the full model and SS_null_ is the sum of squares for the response in the null model, as calculated by the *pgls* function (Orme et al., 2018). The null model included only the intercept but used the same correlation structure as the full model. Additionally, to assess variable importance, we calculated the sum of model probabilities containing each variable (Burnham & Anderson, 2002). Finally, we estimated model‐averaged coefficients and confidence intervals and built 95% confidence model sets by retaining models with a cumulative probability up to 0.95. Models were built using the R package “MumIn” with function *dredge*, and *model*.*avg* for computing model‐averaged estimates (Bartón, 2018).

Predictors were assessed for multicollinearity using generalized variance inflation factors (GVIF). To allow for comparisons between continuous and categorical variables with multiple levels, we calculated a dimensionality correction for categorical variables (Fox & Monette, 1992):

$${\text{GVIF}}^{\frac{1}{\left(2\times \mathrm{df}\right)}}$$

where GVIF is the generalized variance inflation factors and *df* is the degrees of freedom of the predictor (number of categories − 1). The square of this result can be evaluated using VIF thresholds (<2: no collinearity, >5: high collinearity).

## 3 RESULTS

Natal dispersal distances were estimated for 103 species, including 51 with sample size >5 that were retained for analyses (Table S1). Of these 51 species, we were able to estimate the wing aspect ratio of 45 (Table S2). Aspect ratio estimates were generated from an average of 3.6 specimens per species. Finally, one species was dropped due to insufficient population size information. In total, 44 species were used to generate comparative models.

**FIGURE 1:**
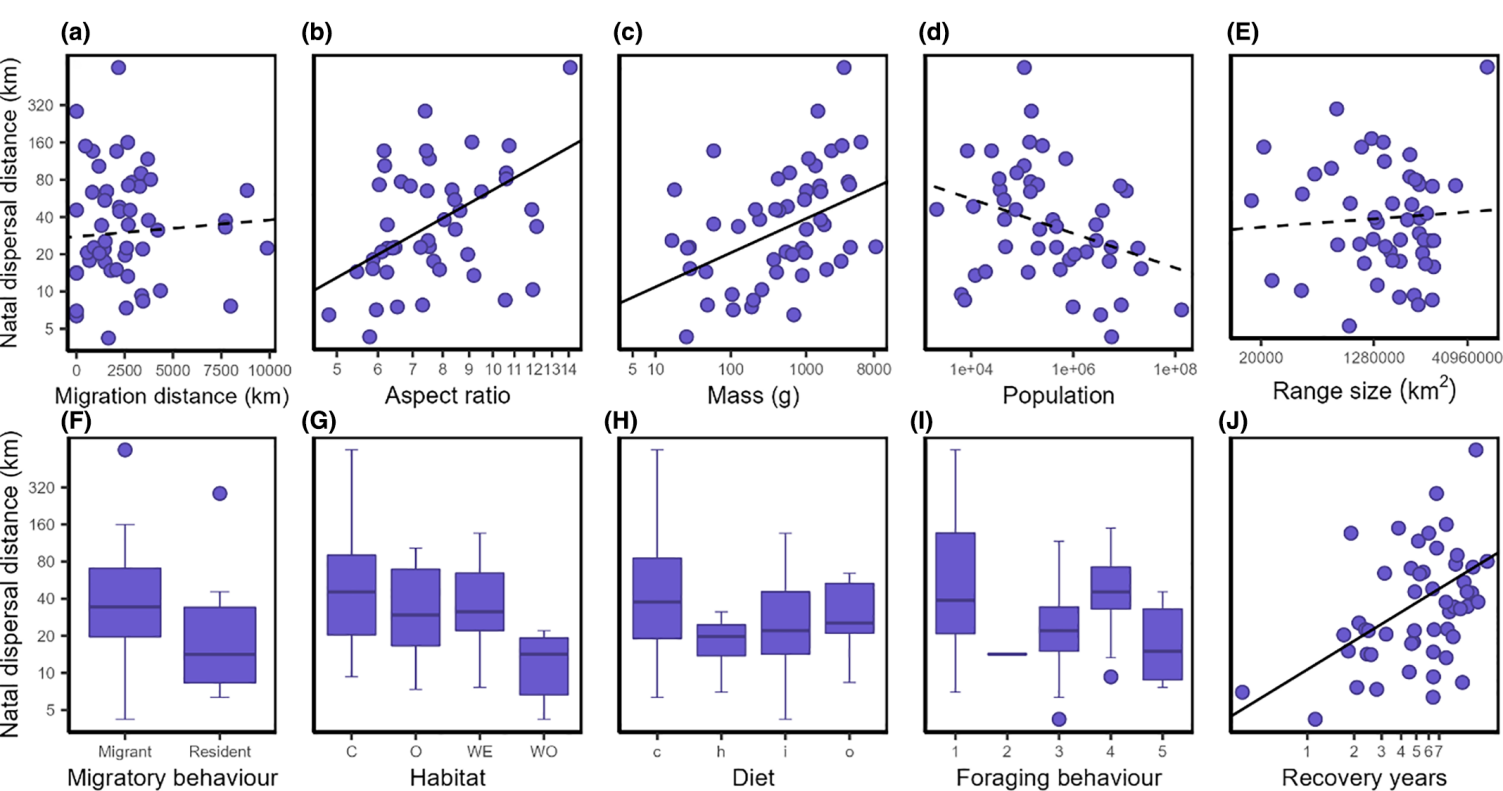
The relationship between natal dispersal distance and 10 morphological, behavioral, and ecological factors in 44 species of North American birds. Lines corresponded to single‐predictor phylogenetic generalized least‐squares models. Solid lines are models whose slopes were significantly different from zero. Habitat categories in G are as follows: coasts (C), open (O), wetlands (WE), and woodlands (WO). Diet categories in H are as follows: carnivores (c), herbivores (h), insectivores (i), and omnivores (o). Foraging behavior categories in I are ranked in increasing flight requirements: surface foraging (1), tree foraging (2), sallying (3), aerial search (4), and aerial capture (5).

In single‐predictor models, the aspect ratio was clearly the best‐performing model based on log‐likelihoods and AIC values (Table 1, Figure 1b). Habitat was the best predictor based on variance explained (*R* ^2^‐values, Table 1). Mean number of years between banding and recovery (recovery year) was positively correlated with dispersal distances (Table 1, Figure 1j). It is the second‐best single‐predictor model, but it achieved low model probability (Table 1). For other predictors, model probability was low. Breeding range, migratory behavior, migration distance, and diet, in particular, were poor predictors of natal dispersal distance (Table 1).

**TABLE 1:** Single‐predictor PGLS models of natal dispersal distance for 44 species of North American birds.

Model	Intercept	Coefficient	*p*‐Value	*df*	*λ*	Log(Lik)	ΔAICc	*p*_model_	*R* ^2^
Aspect ratio	−0.73	2.12 ± 1.56	.0087	2	0.33	−62.7	0	.94	.15
Recovery years	2.37	0.78 ± 0.43	.0007	2	0.42	−66.2	7.04	.03	.21
Population size	4.98	−0.11 ± 0.13	.0871	2	0.31	−66.9	8.47	.01	.06
Habitat	4.23		.0027	4	0.40	−64.7	8.61	.01	.26
Mass	1.81	0.28 ± 0.17	.0016	2	0.00	−68.3	11.23	.00	.19
Foraging behavior	3.84		.1171	5	0.36	−68.3	18.36	.00	.15
Migration behavior	3.56		.4351	2	0.39	−71.9	18.53	.00	.01
Migration distance	3.23	0.04 ± 0.12	.4895	2	0.41	−72.0	18.63	.00	.01
Range	3.16	0.02 ± 0.16	.7778	2	0.47	−72.2	19.02	.00	<.00
Diet	3.85		.2649	4	0.42	−70.1	19.49	.00	.08

*Note*: Aspect ratio, population size, body mass, migration distance, geographic range size, and mean years between banding and recovery were log transformed. For continuous predictors, *p‐*values correspond to regression coefficients; for categorical predictors, *p‐*values correspond to a comparison with a null (for details on coefficients for each level, see Tables S8–S11). *λ* estimates the degree of phylogenetic non‐independence in residuals on a scale from 0 to 1. Log(Lik) is the log likelihood, ΔAICc is the difference between the AICc of the best model and the given model, *p*model is the model probability, and *R* ^2^ is the coefficient of determination.

**FIGURE 2:**
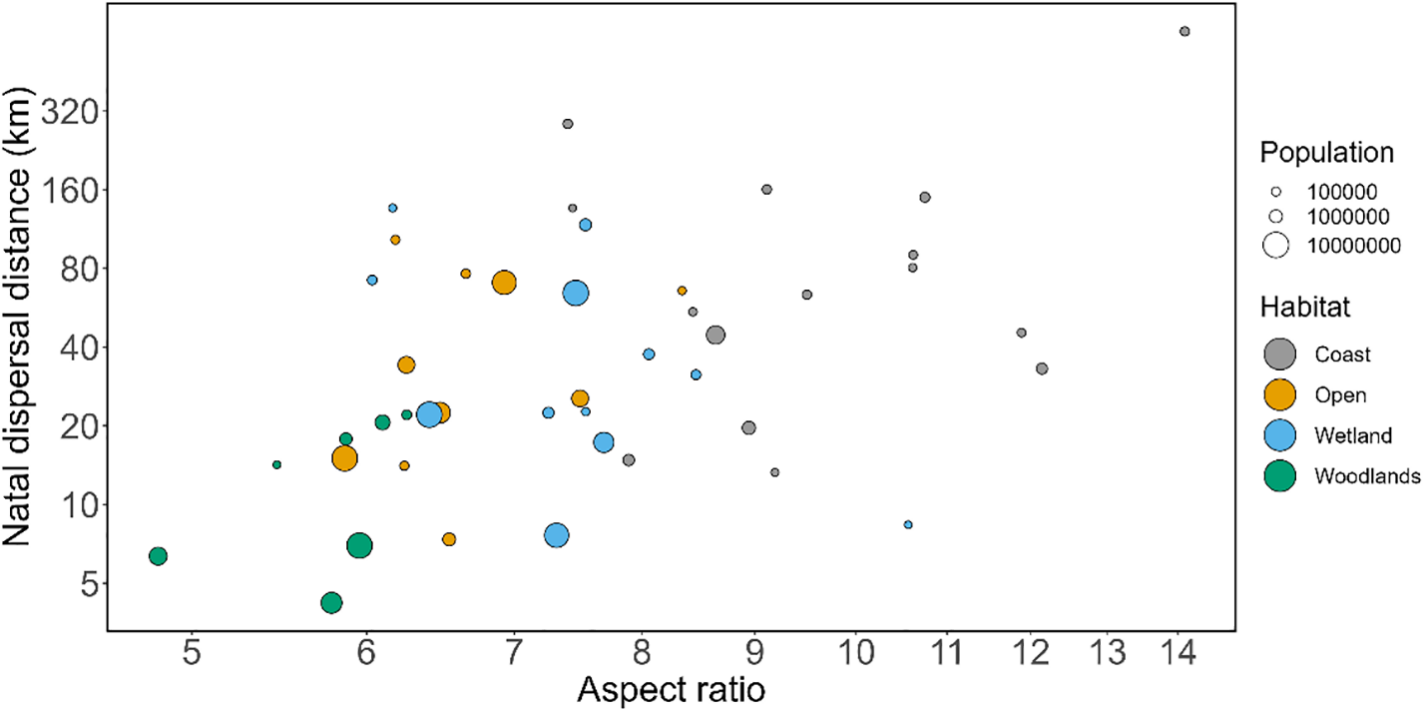
Relationship among flight efficiency (wing aspect ratio), population size, habitat, and natal dispersal distance for 44 species of North American birds. Dot size is proportional to population size up to 100 million individuals (species with greater populations are depicted with the same sized point). Dot color indicates habitat: coasts (gray), open (yellow), wetlands (blue), and woodlands (green).

Among multi‐predictor models, the top model contained aspect ratio, population size, and habitat, and explained 40% of the variation (Figure 2, Table S3). Most habitat categories were significantly different from the reference level (coast) in the best model (Table S4) and the overall effect of habitat was statistically significant (Likelihood ratio test: *p*‐value = .010). The residual phylogenetic inertia was *λ* = 0.50 for this model, indicating that phylogenetically structured variation is still present in model residuals. However, even the best model attained low model probability, it was followed by multiple models with similar fit (ΔAIC < 2), and the 95% confidence model set contained 109 models revealing considerable model uncertainty.

**FIGURE 3:**
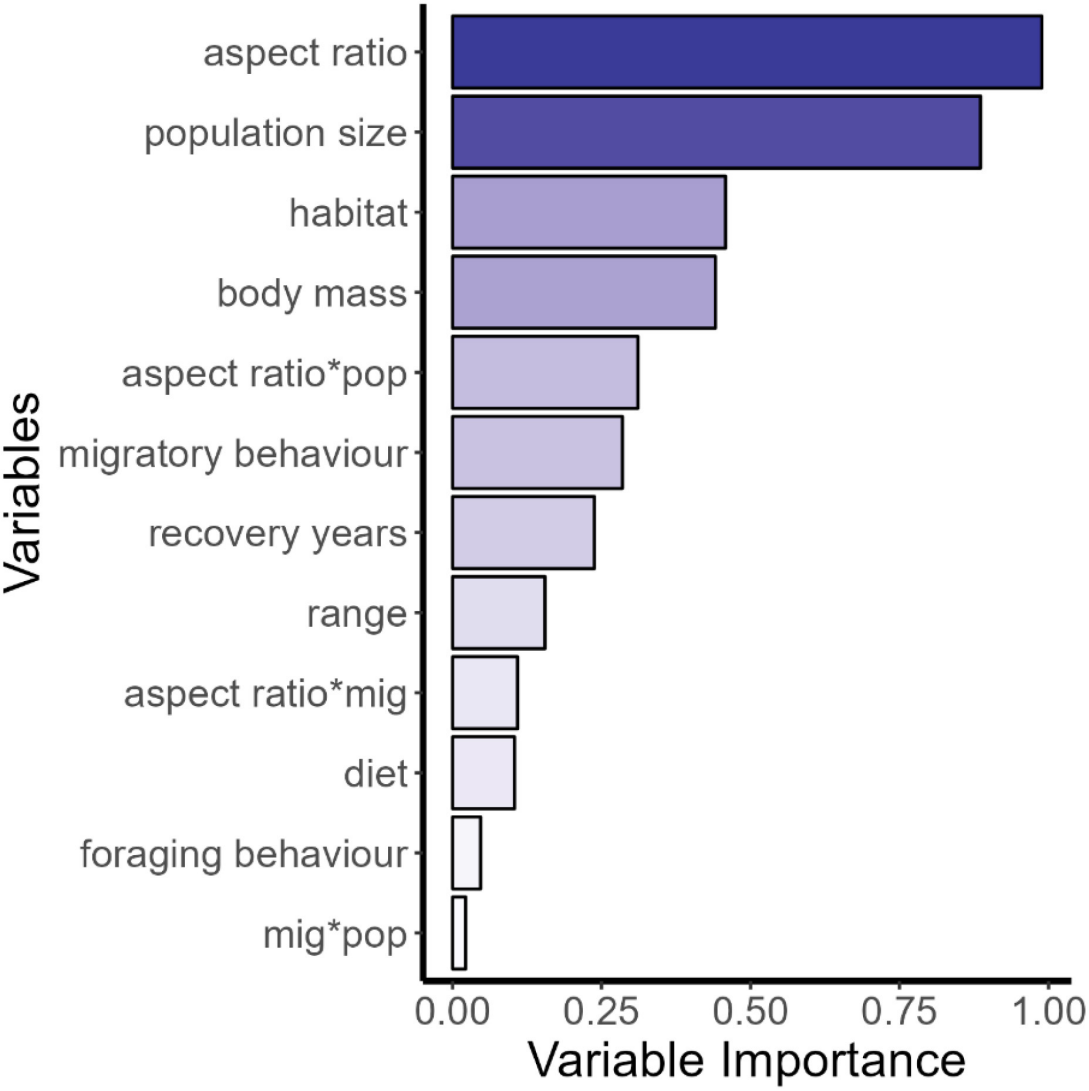
Variable importance from multi‐predictor PGLS models explaining natal dispersal distance among 44 species of North American birds. Variable importance was calculated as the sum of the probabilities of the models containing the given variable.

All models within 5 AIC units of the best model contained aspect ratio. Population size also occurred frequently among these models, whereas other ecological variables occurred in the best models inconsistently. This fact is reflected in the estimated variable importance, which indicated that the aspect ratio was the most important variable (0.99) followed by population size (0.89, Figure 3). In contrast, although appearing in the best model, habitat had lower importance (0.46, Figure 3) and all other predictors were unimportant. (Figure 3). Most model‐averaged estimates had wide confidence intervals that include zero (Table S5).

Inspection of bivariate plots showed no strong colinear relationships among predictors (Figures S1 and S2). However, generalized variance inflation factors (GVIF) suggested that there is moderate multicollinearity involving the aspect ratio and recovery years (Table S6).

## 4 DISCUSSION

We successfully estimated natal dispersal distances for 51 North American bird species. Our method, adapted from Paradis et al. (1998), produced dispersal estimates that are standardized, comparable, and easily replicated. These data add to the growing body of dispersal distances estimated from banding data (Martin & Fahrig, 2018; Paradis et al., 1998; Weeks et al., 2022) and will facilitate future comparative analysis. Because of the number of years elapsed between banding and recovery, some breeding dispersal events may have been added, producing an overestimation of natal dispersal distances. This may limit the use of these estimates for applications in conservation or management that require accurate natal dispersal distances. However, this bias did not obscure the effect of biologically relevant factors on the dispersal process in our analysis.

Our results suggest that flight efficiency, population size, and to a lesser extent, habitat influence natal dispersal distance in this group of North American birds. Only flight efficiency and population size consistently appear in the models that better predicted natal dispersal distances, and thus, only these two factors had high variable importance. Model‐averaged parameter estimates had wide confidence intervals, but this uncertainty could be explained by moderate levels of multicollinearity increasing variance in parameter estimates. Additionally, residual phylogenetic inertia was moderate in our natal dispersal models suggesting that there are additional phylogenetically structured factors influencing dispersal distances. These may include taxon‐dependent biases in dispersal estimates; for example, gulls and terns showed shorter dispersal distances than what their high aspect ratio wings would predict, perhaps as a result of strong philopatric tendencies in these species (Figure S3). Moderate levels of multicollinearity suggest that natal dispersal distances may be influenced by an aspect of a bird's biology that is correlated strongly with flight efficiency, population size, and habitat. We propose that this aspect is the degree of mobility of the species.

The energetic cost of locomotion is expected to influence dispersal distances, but empirical evidence supporting this prediction is scarce (Bonte et al., 2012; Matthysen, 2012). In birds, because flight is a prominent mode of locomotion and is energetically intense (Norberg, 1990; Rayner, 1988), flight efficiency may have a strong influence on dispersal distances (Claramunt, 2021). A comparative analysis of British birds found that flight efficiency, as estimated from wing morphology, was the main factor explaining variation in natal dispersal distances (Claramunt, 2021). We found that flight efficiency is also the main factor influencing dispersal distances among this group of North American birds, independently confirming this prediction in a different continent, and suggesting that this pattern may be general across all birds.

The importance of flight efficiency, as estimated from wing morphology, in determining dispersal distances in birds supports the already prominent use of wing morphology as a proxy for dispersal ability in ecology and evolution (Claramunt et al., 2012; Sheard et al., 2020; Tobias et al., 2020; Weeks et al., 2022). Like in British birds (Claramunt, 2021), we found that more precise estimates of flight efficiency, such as the aspect ratio and the lift‐to‐drag ratio, resulted in better predictions of natal dispersal distance (see Appendix, Table S8). The lower performance of the hand‐wing index can be explained by the fact that it only estimates the elongation of the manual portion of the wing, ignoring variation in arm length, thus underestimating the wing elongation of birds with long arms such as albatrosses, and overestimating wing elongation of birds with short arms such as swifts and hummingbirds (Claramunt & Wright, 2017).

Because of its role in determining the efficiency of movements through space, flight efficiency can also influence other aspects of birds' biology. For example, flight efficiency has a strong influence on the species' ability to cross habitat gaps and move across fragmented landscapes (Claramunt et al., 2012, 2022; Hartfelder et al., 2020; Ibarra‐Macias et al., 2011; Naka et al., 2022). Flight efficiency may also be linked to foraging behavior and, by extension, to diet and habitat (Norberg, 1990; Rayner, 1988; Sherry, 2016). For example, forest‐dwelling birds tend to have low aspect ratio wings and move short distances from tree to tree, whereas coastal birds have high aspect ratio wings and high flight efficiency to endure long flights over water. Therefore, it is likely that the covariation between wing shape and these ecological characteristics is driven by adaptations to different levels of mobility and the degree of development of an aerial lifestyle (Weeks et al., 2022). Because the aspect ratio is a strong predictor of long‐distance locomotor efficiency in birds (Claramunt & Wright, 2017; Norberg, 1990; Pennycuick, 2008), it should closely reflect adaptations for mobility, resulting in a strong signal of multicollinearity with ecological and behavioral factors related to the use of space.

Adaptations for mobility and the distribution of resources may explain the correlation between habitat and dispersal distances. Species in highly productive habitats such as forests disperse shorter distances compared to species in habitats of lower productivity such as grasslands and deserts (open habitats). Freshwater wetlands are highly productive but are sparsely distributed, or linear (rivers); therefore, many wetland species may still require moderate‐to‐high levels of mobility. Coastal habitats are exploited by highly mobile species because of the mobility and limited accessibility of oceanic prey or for commuting to appropriate roosting and breeding sites along the coast (Davoren et al., 2003; Preston, 1990). Therefore, the effect of habitat on dispersal distances might also be mediated by the movement needs of the species. For that reason, once flight efficiency is taken into account, habitat ceases to be an important variable in explaining dispersal distances.

Adaptations for mobility can also explain the negative correlation between natal dispersal distance and population size: species with larger populations exhibited shorter natal dispersal distances. This result reveals that this pattern is not unique to the British avifauna (Claramunt, 2021; Paradis et al., 1998) but may be more general. Population size may be negatively related to dispersal distance because species that use widespread and rich resources or habitats are abundant and do not need to disperse much to find resources (Paradis et al., 1998). On the other hand, species such as raptors or oceanic birds that exploit resources that are sparsely distributed show both low population densities and adaptations for mobility and flight efficiency, which indirectly result in long dispersal distances.

Intraspecific attraction and mating opportunities may also explain the negative relationships between population sizes and dispersal distances independently from the distribution of other resources. Higher conspecific density increases mating opportunities and may decrease the need to move farther for mating and breeding (Doligez et al., 2003; Stamps, 1994; Wagner, 1993). But density itself should be controlled by resource availability, and the fact that this correlation is also intertwined with flight efficiency suggests that species adaptations to mobility are also involved (Bowman, 2003; Claramunt, 2021; Stephens et al., 2019). A caveat to these explanations is the fact that the population estimates used in this analysis represent entire continental breeding populations and may not reflect local population densities. More research is warranted to unpack this pattern between population size and dispersal distance.

Given that levels of mobility required by the distribution of resources may explain variation in dispersal distances, it is surprising that foraging behavior did not exhibit the expected pattern of increased dispersal distances with increased amount of flight required for foraging (Figure 1j). Surface foragers, the birds that in theory require the least amount of flight on the foraging spectrum, showed unexpectedly high dispersal distances. Although some surface foragers such as ducks and shorebirds do not fly during foraging, they are highly mobile in order to reach appropriate foraging grounds. Surface foraging is also often associated with unproductive habitats that lack dense and tall vegetation. In these habitats, species need larger foraging areas and thus enhanced mobility in order to obtain sufficient resources. Finally, even in productive tropical forests, ground‐foraging species may show higher levels of mobility than species that forage on understory vegetation (Claramunt et al., 2022; Naka et al., 2022). These factors may complicate the relationship between foraging behavior and dispersal distances.

We found that migratory behavior was a poor predictor of dispersal distance (Table 1). British birds showed a strong effect of migratory behavior on dispersal distance (Dawideit et al., 2009; Paradis et al., 1998), and both theory and empirical evidence suggest migratory behavior and migration distances depend on long‐distance flight efficiency (Chu et al., 2022; Minias et al., 2015; Norberg, 1990; Nowakowski et al., 2014; Vágási et al., 2016; Vincze et al., 2019). However, other lines of evidence suggest that migration and dispersal may be completely decoupled. Despite the apparently strong effect of migration on dispersal among British birds, most migratory species showed a trend of increasing dispersal distances with increasing flight efficiency that was very similar to the one shown by non‐migratory species (Claramunt, 2021). Long‐distance migration is made possible by unique physiological and behavioral adaptations such as fat accumulation and stopover strategies that may increase flight capabilities beyond the normal capabilities of species during non‐migratory periods (Butler, 2016; Pennycuick, 2008; Winkler et al., 2016). In addition, strong philopatric tendencies among migrants (Winger et al., 2019; Winkler et al., 2016) may minimize and practically nullify any potential effect of migratory movements on dispersal distance. In sum, migratory movements and dispersal movements in birds may be more dissociated than usually assumed and more research is needed on this topic.

We did not find an association between dispersal distances and geographic range size. Despite clear theoretical expectations and some empirical evidence (Alzate & Onstein, 2022; Arango et al., 2022; Capurucho et al., 2020; Laube et al., 2012; Lester et al., 2007), we found that natal dispersal distances of widespread species were similar to those of range‐restricted species. A potential correlation between geographic range size and dispersal distances may be dampened by the negative correlation between dispersal distances and population size, as widespread species tend to be more abundant (Bock & Ricklefs, 1983; Brown & Munger, 1985) and less dispersive (Claramunt, 2021, this study). Further research on the interrelationships among dispersal, abundance, and geographic range size is warranted.

## 5 CONCLUSION

We found that natal dispersal distances depend on the species' flight efficiency and are also correlated with population size. We propose that the interrelationship among flight efficiency, population size, and dispersal can be explained by adaptations to different levels of mobility related to resource density and distribution. We highlight the importance of flight efficiency as a determinant of dispersal distance and find support for the use of wing morphology to infer dispersal ability in birds. The relationship between flight efficiency and dispersal ability in birds provides a framework to explore broader ecological and evolutionary phenomena associated with dispersals such as community connectivity and the interrelationships among geographic distribution, dispersal, and abundance. It may also be helpful in assessing targets for conservation because how birds respond to climate change and habitat fragmentation may depend on their dispersal capabilities (Claramunt et al., 2022; Desrochers, 2010; Martin et al., 2017; Tingley et al., 2009). Despite dispersal's far‐reaching effects on ecology and evolution, there is still much to be learned about its causal factors and macroecological consequences.

## AUTHOR CONTRIBUTIONS

**Jonathan J. Chu:** Conceptualization (equal); data curation (lead); formal analysis (lead); investigation (equal); methodology (equal); project administration (equal); writing – original draft (lead); writing – review and editing (equal). **Santiago Claramunt:** Conceptualization (equal); data curation (supporting); formal analysis (supporting); funding acquisition (lead); investigation (equal); methodology (equal); project administration (equal); supervision (lead); writing – review and editing (equal).

## ACKNOWLEDGEMENT

We would like to acknowledge that the data used in this study were collected by the efforts of many volunteer bird banders for the North American Bird Banding Program administered by Canada's Bird Banding Office and the United States Bird Banding Laboratory. We thank Danny Bystrak and Véronique Drolet‐Gratton of the BBL and BBO respectively for handling data requests and providing early advice on banding data usage. We thank Marie‐Josée Fortin, Jason Weir, Luke Mahler, Njal Rollinson, Mark Peck, Oliver Haddrath, Talia Lowi‐Merri and Hellen Fu for the helpful discussions and advice on early versions of this study. We acknowledge the support of the Natural Sciences and Engineering Research Council of Canada (NSERC), Discovery Grant RGPIN‐2018‐06747.

## CONFLICT OF INTEREST STATEMENT

The authors declare no conflict of interest.

## Supporting information


Appendix S1.

## Data Availability

Data are available in the Dryad Digital Repository https://doi.org/10.5061/dryad.15dv41p1x (Chu & Claramunt, 2023).

## APPENDIX A

**Evaluation of alternative flight efficiency proxies**

We calculated three proxies for flight efficiency: hand‐wing index, aspect ratio, and lift‐to‐drag ratio (Claramunt & Wright, 2017). The hand‐wing index is a proxy for the aspect ratio of the hand portion of the wing that can be measured on traditional museum specimens (Claramunt et al., 2012). The hand‐wing index is defined as:

$$100∙\frac{\left(L_{w}-S_{1}\right)}{L_{w}}$$

where *L*_*w*_ is the distance between the carpal joint and the tip of the longest primary feather, and *S*_*1*_ is the distance between the carpal joint and the tip of the first secondary feather. Hand‐wing indices for all species were obtained from Sheard et al. (2020).

The aspect ratio is the morphological characteristic of the wing that is most influential in determining long‐distance flight efficiency (Norberg, 1990; Pennycuick, 2008). It is calculated as:

$$\frac{B^{2}}{A_{\mathrm{tot}}}$$

where *B* is the wingspan and *A*_tot_ is the total wing area (the area of both wings plus the area of the body between the wings) estimated as:

$$A_{\mathrm{tot}}=2A_{w}+C_{r}∙\left(B-2E\right)$$

in which *A*_*w*_ is the area of a single wing, *C*_*r*_ is the width of wing at the base (known as the root chord), and *E* is the wing extent, measured as the distance from the base to the tip of the longest primary feather perpendicular to *C*_*r*_ (see Claramunt & Wright, 2017; Pennycuick, 2008 for illustrations and further details).

Lift‐to‐drag ratio is the ratio of the lift force that counteracts the body weight to the drag forces during horizontal steady flight, and it is given by the formula:

$$\frac{\mathrm{mgV}}{P}$$

where *m* is body mass, *g* is the gravitational acceleration, *V* is the forward velocity, and *P* is the power required for flight. *P* can be estimated from aerodynamic models of avian flight and morphological variables (Claramunt & Wright, 2017; Pennycuick, 2008). The three main components of the mechanical power required need three morphological measurements: wingspan, wing area, and body mass.

Wingspan and wing area measurements were taken from spread wing images and museum specimen records (further details outlined in main text). Body masses were obtained from Tobias and Pigot (2019), which were based on Dunning (2007). As empirical field measurements of flight velocity are not available for most species, we estimated the maximum lift‐to‐drag ratio for each species by maximizing it with respect to velocity using function *optim* in R 4.0 (R Core Team, 2019). This approach is based on evidence suggesting that birds tend to fly at velocities that maximizes their lift‐to‐drag ratio (known as maximum‐range velocities) during migration or commuting (Bruderer & Boldt, 2001; Pennycuick, 1997; Pennycuick et al., 2013). For the estimation of powers, we assumed an induced power factor = 1, a body drag coefficient = 0.1, and estimated the body's frontal area from the body mass as 0.01 m^2/3^ following Pennycuick (2008), Pennycuick et al. (2013), and Claramunt and Wright (2017).

Aspect ratio and lift‐to‐drag and hand‐wing indexes were used as predictors of natal dispersal distances using phylogenetic least squares models (PGLS).

All three flight efficiency proxies were positively and significantly correlated with dispersal distance. Aspect ratio was the best proxy in terms of both models' fit, AIC, and explained variance (Table S7). The lift‐to‐drag ratio was the second‐best proxy, only slightly underperforming in fit, AIC, and explained variance. The hand‐wing index had the lowest performance of the three proxies (Table S7). Similar results were found among British birds, in which the wing's aspect ratio and the lift‐to‐drag ratio outperformed the hand‐wing index (Claramunt, 2021).
