# Supplementary material for: Determinants of natal dispersal distances in North American birds
Source: Ecol Evol. 2023 Feb 8;13(2):e9789. doi: 10.1002/ece3.9789 (PMC9909001; doi:10.1002/ece3.9789)
Supplement: Supplementary file 1 — Appendix S1. [file ECE3-13-e9789-s001.docx]

# **Supplementary Information**

**Supplementary Figures**


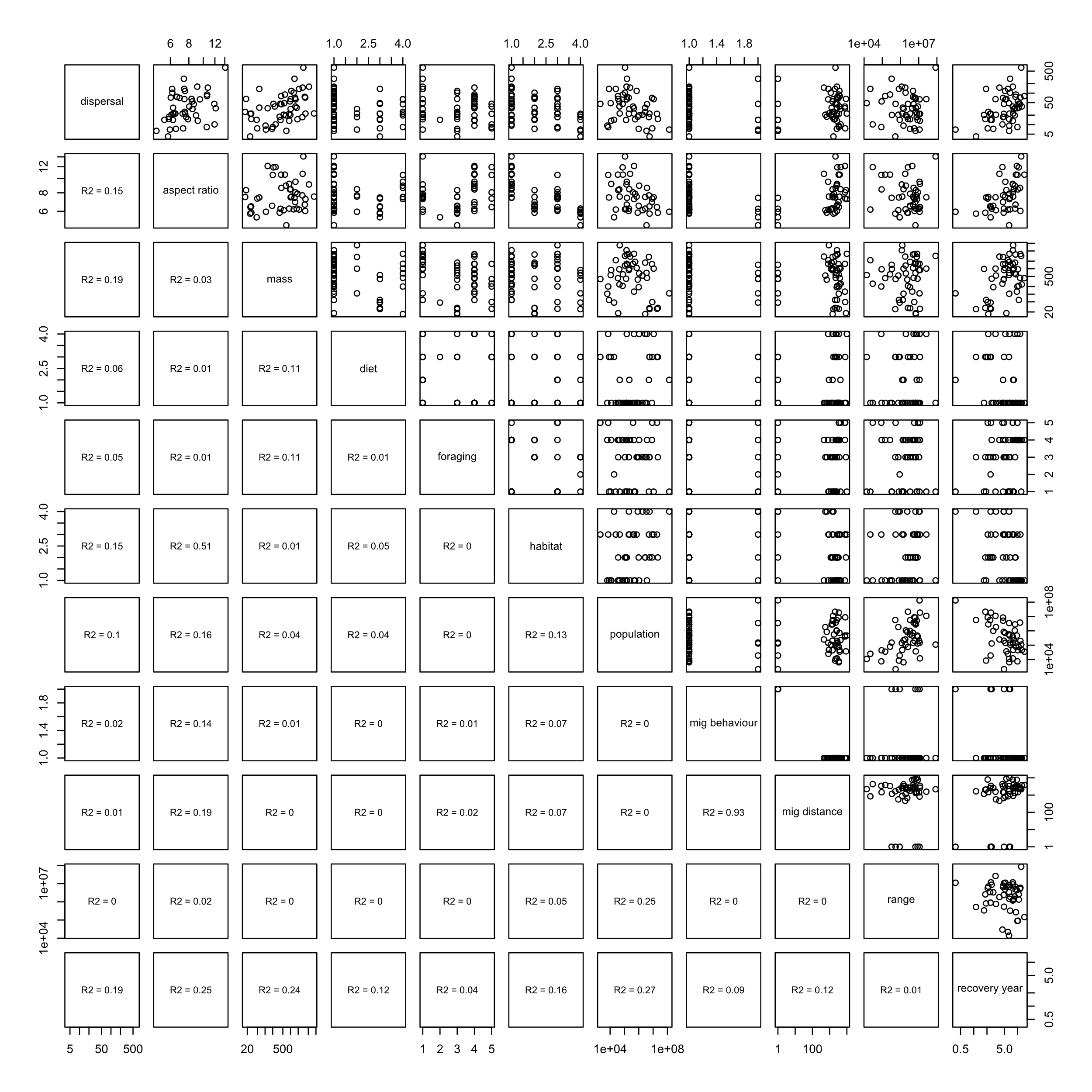


Figure S1.

Plots of all variables included in dispersal distance models analyzing determiannts of natal dispersal distances in 44 species of North American birds. Three variables are categorical: diet, foraging (foraging behaviour), and habitat. The rest are continuous and natural log-transformed: dispersal (natal dispersal distance in km), aspect ratio, body mass (in g), mig distance (migration distance), range (breeding geographic range size) or binary, mig. behaviour (migratory behaviour). All pairs of variables are plotted as scatterplots above the diagonal, correlation coefficients are shown below the diagonals.


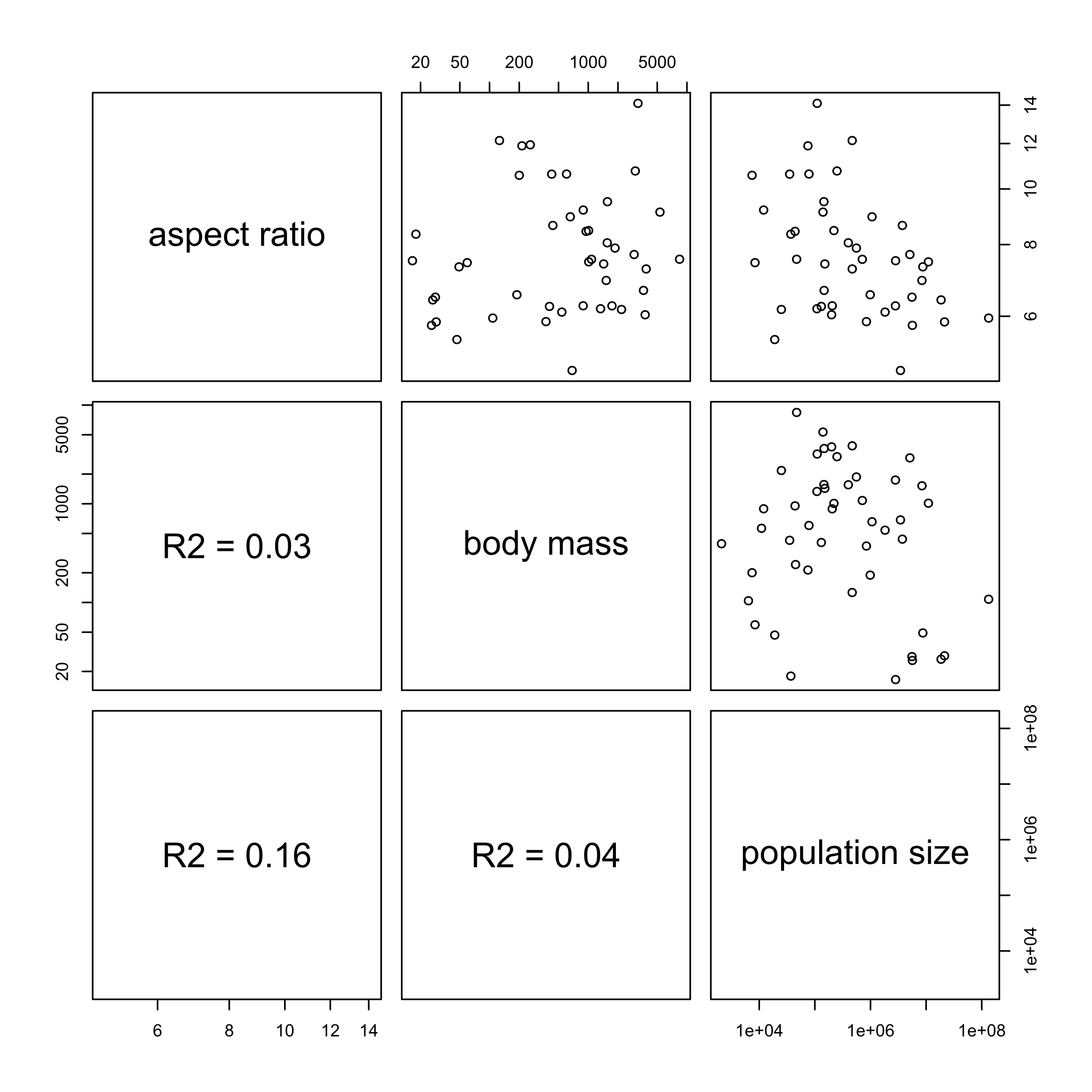


Figure S2. Pairs plot of major continuous independent parameters included in dispersal distance models: AR (aspect ratio), body mass, and population size. All pairs of variables are plotted as scatterplots above the diagonal, correlation coefficients are shown below the diagonals.


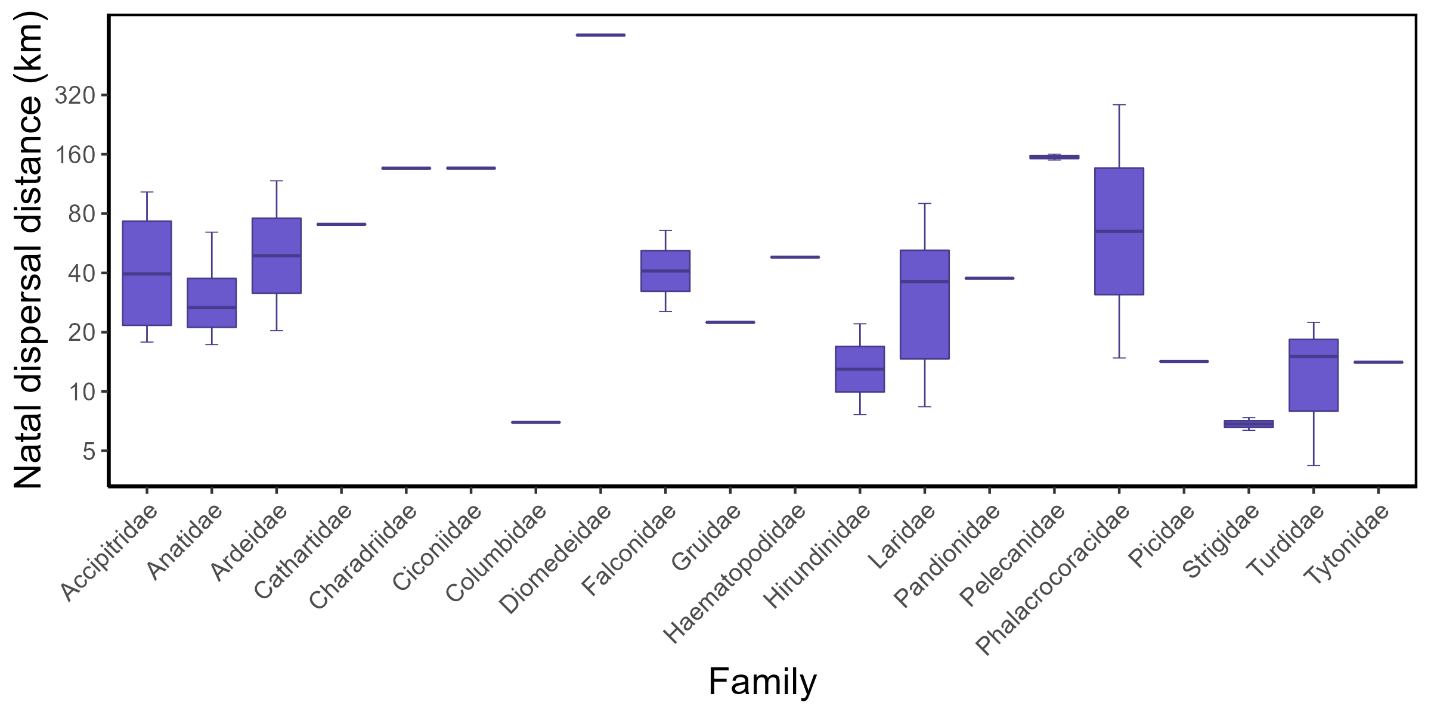
Figure S3.

Boxplots of natal dispersal distances by family. Boxes represent interquartile range, with the median represented by a darker line within the box. Families with a single species in the dataset are represented by a single line. Whiskers represent maximum and minimum values within 1.5 times the interquartile range.

**Supplementary Tables**

Table S1.

Natal dispersal distances estimated for 103 species of North American birds using banding data. *n* is the sample size, the number of records that satisfied filtering criteria. Also shown is the mean number of years between banding and recovery for each species as well as the range; these values are only calculated for species with n > 4 as those were the ones used in further analyses.

| Scientific name | Geometric mean natal dispersal distance (km) | n | Mean recovery time (years) | Range of recovery time (years) | Min recovery time (years) | Max recovery time (years) |
| --- | --- | --- | --- | --- | --- | --- |
| *Branta canadensis* | 17.30 | 940 | 4.66 | 18 | 2 | 20 |
| *Progne subis* | 7.63 | 690 | 2.08 | 8 | 1 | 9 |
| *Cathartes aura* | 70.59 | 435 | 4.63 | 9 | 2 | 11 |
| *Falco peregrinus* | 65.67 | 424 | 5.54 | 19 | 1 | 20 |
| *Sterna dougallii* | 9.29 | 377 | 6.43 | 21 | 2 | 23 |
| *Pandion haliaetus* | 37.54 | 154 | 7.74 | 21 | 3 | 24 |
| *Hydroprogne caspia* | 90.05 | 130 | 9.13 | 30 | 2 | 32 |
| *Pelecanus occidentalis* | 149.54 | 128 | 3.84 | 17 | 1 | 18 |
| *Thalasseus maximus* | 80.45 | 124 | 14.23 | 29 | 2 | 31 |
| *Nannopterum auritum* | 14.80 | 120 | 6.43 | 21 | 1 | 22 |
| *Haliaeetus leucocephalus* | 72.14 | 109 | 11.54 | 27 | 5 | 32 |
| *Cygnus buccinator* | 22.64 | 106 | 7.91 | 18 | 2 | 20 |
| *Pelecanus erythrorhynchos* | 160.21 | 92 | 7.78 | 16 | 3 | 19 |
| *Tachycineta bicolor* | 22.06 | 84 | 2.46 | 6 | 1 | 7 |
| *Falco sparverius* | 25.45 | 77 | 2.14 | 6 | 1 | 7 |
| *Tyto alba* | 14.07 | 75 | 2.56 | 11 | 1 | 12 |
| *Zenaida macroura* | 6.97 | 73 | 0.38 | 3 | 0 | 3 |
| *Phoebastria nigripes* | 644.17 | 68 | 12.06 | 21 | 5 | 26 |
| *Sterna hirundo* | 33.07 | 62 | 9.58 | 21 | 2 | 23 |
| *Thalasseus sandvicensis* | 45.40 | 59 | 10.58 | 20 | 3 | 23 |
| *Larus argentatus* | 13.27 | 50 | 7.76 | 25 | 4 | 29 |
| *Anas platyrhynchos* | 64.38 | 46 | 3.13 | 10 | 1 | 11 |
| *Sternula antillarum* | 34.60 | 43 | 10.63 | 20 | 2 | 22 |
| *Sialia sialis* | 15.04 | 36 | 1.83 | 4 | 1 | 5 |
| *Mycteria americana* | 136.08 | 25 | 6.00 | 17 | 3 | 20 |
| *Haematopus palliatus* | 19.79 | 25 | 6.36 | 9 | 3 | 12 |
| *Sialia currucoides* | 22.44 | 22 | 2.36 | 4 | 1 | 5 |
| *Charadrius melodus* | 136.00 | 21 | 1.90 | 6 | 1 | 7 |
| *Buteo jamaicensis* | 34.22 | 20 | 8.75 | 28 | 1 | 29 |
| *Rynchops niger* | 10.17 | 18 | 4.50 | 16 | 2 | 18 |
| *Rostrhamus sociabilis* | 45.54 | 18 | 4.89 | 11 | 1 | 12 |
| *Larus occidentalis* | 54.53 | 17 | 10.12 | 15 | 4 | 19 |
| *Urile penicillatus* | 285.52 | 15 | 6.73 | 14 | 2 | 16 |
| *Accipiter cooperii* | 17.81 | 15 | 4.80 | 12 | 1 | 13 |
| *Aquila chrysaetos* | 76.30 | 14 | 8.93 | 23 | 3 | 26 |
| *Athene cunicularia* | 7.36 | 14 | 2.79 | 6 | 1 | 7 |
| *Ardea alba* | 117.36 | 13 | 5.15 | 17 | 1 | 18 |
| *Larus marinus* | 63.44 | 12 | 5.25 | 4 | 4 | 8 |
| *Larus delawarensis* | 44.50 | 12 | 11.33 | 19 | 2 | 21 |
| *Dryobates borealis* | 14.21 | 12 | 2.42 | 4 | 1 | 5 |
| *Larus californicus* | 19.64 | 10 | 8.60 | 25 | 2 | 27 |
| *Antigone canadensis* | 22.43 | 10 | 6.50 | 16 | 2 | 18 |
| *Gelochelidon nilotica* | 8.37 | 8 | 9.88 | 16 | 4 | 20 |
| *Buteo regalis* | 102.94 | 8 | 6.75 | 12 | 2 | 14 |
| *Sialia mexicana* | 4.21 | 8 | 1.13 | 1 | 1 | 2 |
| *Thalasseus elegans* | 37.70 | 7 | 12.43 | 7 | 9 | 16 |
| *Branta bernicla* | 31.34 | 7 | 8.14 | 18 | 2 | 20 |
| *Egretta rufescens* | 20.35 | 7 | 1.71 | 4 | 1 | 5 |
| *Accipiter gentilis* | 22.03 | 7 | 4.86 | 10 | 2 | 12 |
| *Buteo lineatus* | 20.62 | 5 | 3.20 | 5 | 1 | 6 |
| *Strix varia* | 6.35 | 5 | 6.40 | 8 | 3 | 11 |
| *Aix sponsa* | 4.38 | 4 |  |  |  |  |
| *Somateria fischeri* | 9.23 | 4 |  |  |  |  |
| *Charadrius nivosus* | 37.10 | 4 |  |  |  |  |
| *Myiarchus cinerascens* | 9.32 | 4 |  |  |  |  |
| *Gavia immer* | 10.43 | 3 |  |  |  |  |
| *Plegadis falcinellus* | 203.36 | 3 |  |  |  |  |
| *Nycticorax nycticorax* | 6.29 | 3 |  |  |  |  |
| *Scolopax minor* | 260.21 | 3 |  |  |  |  |
| *Bubo scandiacus* | 3.55 | 3 |  |  |  |  |
| *Hirundo rustica* | 16.96 | 3 |  |  |  |  |
| *Alca torda* | 477.46 | 2 |  |  |  |  |
| *Sterna paradisaea* | 44.51 | 2 |  |  |  |  |
| *Anser caerulescens* | 511.42 | 2 |  |  |  |  |
| *Egretta thula* | 42.63 | 2 |  |  |  |  |
| *Buteo swainsoni* | 14.74 | 2 |  |  |  |  |
| *Falco mexicanus* | 114.56 | 2 |  |  |  |  |
| *Falco columbarius* | 10.37 | 2 |  |  |  |  |
| *Bubo virginianus* | 10.25 | 2 |  |  |  |  |
| *Corvus corax* | 3.37 | 2 |  |  |  |  |
| *Sturnus vulgaris* | 17.14 | 2 |  |  |  |  |
| *Passer domesticus* | 2.78 | 2 |  |  |  |  |
| *Cepphus columba* | 41.47 | 1 |  |  |  |  |
| *Uria aalge* | 19.11 | 1 |  |  |  |  |
| *Uria lomvia* | 1500.85 | 1 |  |  |  |  |
| *Rissa tridactyla* | 25.24 | 1 |  |  |  |  |
| *Leucophaeus atricilla* | 14.04 | 1 |  |  |  |  |
| *Sterna forsteri* | 552.67 | 1 |  |  |  |  |
| *Chlidonias niger* | 68.51 | 1 |  |  |  |  |
| *Hydrobates furcatus* | 5.64 | 1 |  |  |  |  |
| *Phaethon rubricauda* | 1499.73 | 1 |  |  |  |  |
| *Sula leucogaster* | 108.52 | 1 |  |  |  |  |
| *Morus bassanus* | 2715.28 | 1 |  |  |  |  |
| *Mergus merganser* | 0.67 | 1 |  |  |  |  |
| *Anas fulvigula* | 57.86 | 1 |  |  |  |  |
| *Anas acuta* | 2.54 | 1 |  |  |  |  |
| *Aythya affinis* | 0.70 | 1 |  |  |  |  |
| *Bucephala clangula* | 1.17 | 1 |  |  |  |  |
| *Somateria spectabilis* | 1.32 | 1 |  |  |  |  |
| *Melanitta deglandi* | 10.81 | 1 |  |  |  |  |
| *Anser canagicus* | 810.11 | 1 |  |  |  |  |
| *Plegadis chihi* | 789.34 | 1 |  |  |  |  |
| *Recurvirostra americana* | 353.97 | 1 |  |  |  |  |
| *Limosa haemastica* | 21.42 | 1 |  |  |  |  |
| *Numenius phaeopus* | 6.91 | 1 |  |  |  |  |
| *Charadrius wilsonia* | 2.40 | 1 |  |  |  |  |
| *Haematopus bachmani* | 22.60 | 1 |  |  |  |  |
| *Corvus brachyrhynchos* | 51.76 | 1 |  |  |  |  |
| *Vireo atricapilla* | 4.54 | 1 |  |  |  |  |
| *Campylorhynchus brunneicapillus* | 0.46 | 1 |  |  |  |  |
| *Troglodytes aedon* | 147.43 | 1 |  |  |  |  |
| *Sitta pusilla* | 3.53 | 1 |  |  |  |  |
| *Poecile atricapillus* | 3.00 | 1 |  |  |  |  |

Table S2.

Parameter estimates for the 51 North American bird species with natal dispersal estimates. AR is aspect ratio, HWI is hand-wing index, LD is lift-to-drag ratio, Mass is body mass, Forage is foraging behaviour, Mig behaviour is migratory behaviour and Mig distance is migratory distance in kilometres.

| Scientific name | Dispersal distance (km) | AR | HWI | LD | Mass (g) | Diet | | | Forage | Habitat | | Population size | Mig behaviour | | Mig distance (km) | | Range size (km^2^) |
| --- | --- | --- | --- | --- | --- | --- | --- | --- | --- | --- | --- | --- | --- | --- | --- | --- | --- |
| *Accipiter cooperii* | 17.81 | 5.87 | 32.26 | 13.97 | 373.10 | | c | 3 | | | WO | 844899 | Migrant | 675.65 | | 2354438 | |
| *Accipiter gentilis* | 22.03 | 6.26 | 36.49 | 15.84 | 888.17 | | c | 3 | | | WO | 205103 | Migrant | 1438.65 | | 10478038 | |
| *Anas platyrhynchos* | 64.38 | 7.46 | 50.78 | 12.88 | 1012.90 | | o | 1 | | | WE | 11000000 | Migrant | 1567.15 | | 26225519 | |
| *Aquila chrysaetos* | 76.30 | 6.66 | 36.97 | 17.51 | 3629.16 | | c | 4 | | | O | 146673 | Migrant | 2869.04 | | 4858709 | |
| *Athene cunicularia* | 7.36 | 6.54 | 28.33 | 16.51 | 189.18 | | c | 3 | | | O | 987921 | Migrant | 2575.75 | | 3306769 | |
| *Branta bernicla* | 31.34 | 8.46 | 52.20 | 15.50 | 1008.61 | | h | 1 | | | WE | 220000 | Migrant | 4207.51 | | 1465813 | |
| *Branta canadensis* | 17.30 | 7.69 | 49.37 | 14.54 | 2916.93 | | h | 1 | | | WE | 5100000 | Migrant | 1481.86 | | 7773080 | |
| *Buteo jamaicensis* | 34.22 | 6.26 | 34.08 | 16.40 | 1738.97 | | c | 3 | | | O | 2808115 | Migrant | 1299.47 | | 6934401 | |
| *Buteo lineatus* | 20.62 | 6.10 | 33.43 | 17.04 | 539.93 | | c | 3 | | | WO | 1827010 | Migrant | 562.62 | | 760138 | |
| *Buteo regalis* | 102.94 | 6.18 | 41.31 | 18.13 | 1332.46 | | c | 3 | | | O | 109004 | Migrant | 1159.76 | | 1895561 | |
| *Ardea alba* | 117.36 | 7.54 | 25.70 | 21.45 | 1079.09 | | c | 3 | | | WE | 712641 | Migrant | 3687.19 | | 4869643 | |
| *Cathartes aura* | 70.59 | 6.93 | 40.70 | 21.37 | 1520.55 | | c | 4 | | | O | 8418387 | Migrant | 3272.85 | | 6457223 | |
| *Charadrius melodus* | 136.00 | 7.44 | 54.46 | 15.03 | 59.38 | | i | 1 | | | C | 8400 | Migrant | 2077.75 | | 816346 | |
| *Cygnus buccinator* | 22.64 | 7.54 | 42.69 | 17.64 | 8419.72 | | h | 1 | | | WE | 47000 | Migrant | 905.56 | | 1255578 | |
| *Egretta rufescens* | 20.35 | NA | 37.19 | NA | 983.93 | | c | 1 | | | C | NA | Migrant | 1183.20 | | 337385 | |
| *Falco peregrinus* | 65.67 | 8.34 | 55.42 | 17.69 | 17.94 | | c | 3 | | | O | 37000 | Migrant | 8831.71 | | 6877026 | |
| *Falco sparverius* | 25.45 | 7.50 | 43.68 | 18.81 | 16.55 | | o | 3 | | | O | 2827776 | Migrant | 1485.96 | | 6870582 | |
| *Antigone canadensis* | 22.43 | 7.25 | 43.74 | 16.50 | 3862.22 | | o | 1 | | | WE | 469538 | Migrant | 9874.74 | | 8186404 | |
| *Haematopus palliatus* | 48.05 | NA | 48.05 | NA | 564.15 | | i | 1 | | | C | 11000 | Migrant | 2221.20 | | 13967 | |
| *Haliaeetus leucocephalus* | 72.14 | 6.04 | 41.38 | 17.64 | 3771.80 | | c | 4 | | | WE | 200000 | Migrant | 2686.38 | | 5895839 | |
| *Larus argentatus* | 13.27 | 9.19 | 55.26 | 24.80 | 889.92 | | c | 4 | | | C | 12000 | Migrant | 2656.38 | | 11667237 | |
| *Larus californicus* | 19.64 | 8.94 | 58.42 | 23.35 | 657.31 | | o | 4 | | | C | 1065791 | Migrant | 2515.21 | | 1970441 | |
| *Larus delawarensis* | 44.50 | 8.64 | 56.25 | 23.10 | 437.89 | | o | 4 | | | C | 3740458 | Migrant | 2232.08 | | 5243636 | |
| *Larus marinus* | 63.44 | 9.50 | 52.20 | 24.21 | 1565.58 | | o | 4 | | | C | 145361 | Migrant | 819.05 | | 11176007 | |
| *Larus occidentalis* | 54.53 | 8.44 | 50.99 | 21.87 | 951.53 | | c | 4 | | | C | 44003 | Migrant | 1465.75 | | 91660 | |
| *Mycteria americana* | 136.08 | 6.17 | 37.02 | 17.16 | 2173.57 | | c | 1 | | | WE | 25000 | Migrant | 846.51 | | 22062 | |
| *Pandion haliaetus* | 37.54 | 8.06 | 42.16 | 21.20 | 1557.48 | | c | 5 | | | WE | 399228 | Migrant | 7720.55 | | 9017418 | |
| *Pelecanus erythrorhynchos* | 160.21 | 9.11 | 44.90 | 23.02 | 5328.60 | | c | 1 | | | C | 140000 | Migrant | 2651.51 | | 1180312 | |
| *Pelecanus occidentalis* | 149.54 | 10.75 | 48.10 | 27.65 | 2998.54 | | c | 4 | | | C | 250000 | Migrant | 466.41 | | 1823609 | |
| *Nannopterum auritum* | 14.80 | 7.89 | 40.73 | 16.82 | 1868.33 | | c | 1 | | | C | 557887 | Migrant | 1776.28 | | 3381971 | |
| *Urile penicillatus* | 285.52 | 7.40 | 37.16 | 15.04 | 1432.75 | | c | 1 | | | C | 151200 | Resident | 1.00 | | 326319 | |
| *Phoebastria nigripes* | 644.17 | 14.10 | 64.68 | 28.32 | 3190.54 | | c | 1 | | | C | 110000 | Migrant | 2176.28 | | 84624701 | |
| *Dryobates borealis* | 14.21 | 5.46 | 32.08 | 13.52 | 46.71 | | i | 2 | | | WO | 19000 | Resident | 1.00 | | 901954 | |
| *Progne subis* | 7.63 | 7.32 | 51.29 | 16.17 | 49.11 | | i | 5 | | | WE | 8728591 | Migrant | 7980.92 | | 5699139 | |
| *Rostrhamus sociabilis* | 45.54 | NA | 39.07 | NA | 393.66 | | i | 5 | | | WE | 2100 | Resident | 1.00 | | 537238 | |
| *Rynchops niger* | 10.17 | 11.94 | 67.42 | 26.68 | 258.57 | | c | 5 | | | C | NA | Migrant | 4345.37 | | 29830 | |
| *Sialia currucoides* | 22.44 | 6.48 | 36.60 | 15.49 | 28.28 | | i | 3 | | | O | 5568751 | Migrant | 2600.94 | | 3417740 | |
| *Sialia mexicana* | 4.21 | 5.78 | 37.24 | 13.68 | 25.85 | | i | 3 | | | WO | 5661189 | Migrant | 1654.70 | | 518561 | |
| *Sialia sialis* | 15.04 | 5.87 | 32.77 | 12.70 | 28.81 | | i | 3 | | | O | 21439020 | Migrant | 2068.98 | | 2569285 | |
| *Sternula antillarum* | 34.60 | NA | 69.13 | NA | 59.37 | | c | 4 | | | C | NA | Migrant | 2683.13 | | 1332740 | |
| *Hydroporgne caspia* | 90.05 | 10.62 | 66.22 | 25.81 | 602.71 | | c | 4 | | | C | 78325 | Migrant | 3340.05 | | 267132 | |
| *Sterna dougallii* | 9.29 | NA | 69.12 | NA | 104.07 | | c | 4 | | | C | 6400 | Migrant | 3371.83 | | 1460062 | |
| *Thalasseus elegans* | 37.70 | NA | 66.60 | NA | 242.07 | | c | 4 | | | C | 45000 | Migrant | 3724.48 | |  | |
| *Sterna hirundo* | 33.07 | 12.15 | 67.26 | 27.54 | 126.06 | | c | 4 | | | C | 468971 | Migrant | 7709.43 | | 4445999 | |
| *Sterna maxima* | 80.45 | 10.61 | 61.37 | 22.20 | 426.32 | | c | 4 | | | C | 35206 | Migrant | 3839.26 | | 144753 | |
| *Sterna nilotica* | 8.37 | 10.56 | 67.04 | 28.16 | 200.12 | | o | 5 | | | WE | 7430 | Migrant | 3443.94 | | 88515 | |
| *Thalasseus sandvicensis* | 45.40 | 11.89 | 69.23 | 24.42 | 213.12 | | c | 4 | | | C | 75060 | Migrant | 2770.85 | | 2558743 | |
| *Strix varia* | 6.35 | 4.83 | 29.56 | 14.55 | 686.39 | | c | 3 | | | WO | 3458782 | Resident | 1.00 | | 6628600 | |
| *Tachycineta bicolor* | 22.06 | 6.41 | 54.34 | 14.21 | 26.60 | | i | 5 | | | WE | 18581775 | Migrant | 3429.38 | | 11720974 | |
| *Tyto alba* | 14.07 | 6.24 | 41.40 | 19.11 | 404.93 | | c | 4 | | | O | 130751 | Resident | 1.00 | | 8680409 | |
| *Zenaida macroura* | 6.97 | 5.96 | 35.14 | 12.59 | 107.82 | | h | 1 | | | WO | 1.33E+08 | Resident | 1.00 | | 11134158 | |

Table S3.

Multi-predictor PGLS models of the relationship between flight efficiency, ecological and behavioural variables and natal dispersal distance for 44 species of North American birds. Three model sets are listed corresponding to the three flight efficiency proxies. Only top 20 models are shown. * indicates interaction terms and main effects. Log(Lik) is the log-likelihood, ΔAICc is the difference between the AICc of the best model, listed at the top, and the given model, *Pmodel* is the model probability, and *R^2^* is the coefficient of determination.

| **Model** | **df** | **Log(Lik)** | **AICc** | **ΔAIC** | ***P_model_*** | ***R^2^*** |
| --- | --- | --- | --- | --- | --- | --- |
| aspect ratio + population + habitat | 6 | -50.6 | 115.4 | 0.00 | 0.09 | 0.40 |
| aspect ratio * population + mass | 5 | -52.4 | 116.4 | 0.97 | 0.05 | 0.38 |
| aspect ratio + population + habitat + mass | 7 | -49.8 | 116.6 | 1.19 | 0.05 | 0.43 |
| aspect ratio + population + mass | 4 | -53.8 | 116.7 | 1.26 | 0.05 | 0.34 |
| aspect ratio * migration + population + mass | 6 | -51.5 | 117.3 | 1.90 | 0.03 | 0.42 |
| aspect ratio + population + habitat + recovery year | 7 | -50.1 | 117.4 | 1.96 | 0.03 | 0.42 |
| aspect ratio * population + habitat | 7 | -50.2 | 117.5 | 2.02 | 0.03 | 0.42 |
| aspect ratio * population | 4 | -54.3 | 117.6 | 2.19 | 0.03 | 0.32 |
| aspect ratio + population + habitat + migration | 7 | -50.3 | 117.8 | 2.32 | 0.03 | 0.41 |
| aspect ratio + population + habitat + range | 7 | -50.5 | 118.0 | 2.62 | 0.02 | 0.41 |
| aspect ratio * population + recovery year | 5 | -53.3 | 118.2 | 2.79 | 0.02 | 0.34 |
| aspect ratio * population + mass + diet | 8 | -49.2 | 118.4 | 3.01 | 0.02 | 0.48 |
| aspect ratio * population + habitat + mass | 8 | -49.2 | 118.6 | 3.16 | 0.02 | 0.45 |
| aspect ratio + population + mass + diet | 7 | -50.8 | 118.8 | 3.33 | 0.02 | 0.44 |
| aspect ratio + population | 3 | -56.1 | 118.8 | 3.41 | 0.02 | 0.25 |
| aspect ratio * population + mass + recovery year | 6 | -52.3 | 118.9 | 3.48 | 0.02 | 0.38 |
| aspect ratio * population + mass + range | 6 | -52.3 | 119.0 | 3.53 | 0.01 | 0.38 |
| aspect ratio * population + mass + migration | 6 | -52.4 | 119.1 | 3.66 | 0.01 | 0.38 |
| aspect ratio + population + mass + range | 5 | -53.8 | 119.2 | 3.79 | 0.01 | 0.34 |
| aspect ratio + population + mass + recovery year | 5 | -53.8 | 119.2 | 3.79 | 0.01 | 0.34 |

Table S4.

Coefficients for the best PGLS multi-predictor-model containing aspect ratio as the flight efficiency proxy. Habitat category “coast” was set as the reference level for the habitat predictor.

|  | **β** | **Standard Error** | **t-value** | **P-value** |
| --- | --- | --- | --- | --- |
| intercept | 0.73 | 0.56 | 1.31 | 0.20 |
| aspect ratio | 0.02 | 0.23 | 0.07 | 0.94 |
| population | -0.15 | 0.15 | -1.00 | 0.32 |
| habitat (open) | -0.93 | 0.54 | -1.73 | 0.09 |
| habitat (wetlands) | -1.03 | 0.44 | -2.36 | 0.02 |
| habitat (woodlands) | -1.89 | 0.64 | -2.98 | <0.01 |
| recovery year | 0.04 | 0.05 | 0.87 | 0.39 |

Table S5.

Model averaged estimates and 95% confidence interval bounds for variables in the aspect ratio model set.

| **Variables** | **Estimate** | | **Standard error** | **95% CI - Lower** | | **95% CI - Upper** |
| --- | --- | --- | --- | --- | --- | --- |
| intercept | | 0.47 | 0.58 | | -0.66 | 1.61 |
| habitat (open) | | -0.41 | 0.59 | | -1.56 | 0.74 |
| habitat (wetland) | | -0.44 | 0.58 | | -1.57 | 0.68 |
| habitat (woodland) | | -0.86 | 1.04 | | -2.90 | 1.18 |
| population | | -0.13 | 0.17 | | -0.46 | 0.20 |
| aspect ratio | | 0.26 | 0.28 | | -0.28 | 0.81 |
| recovery year | | 0.01 | 0.04 | | -0.06 | 0.09 |
| aspect ratio:population | | 0.09 | 0.17 | | -0.25 | 0.43 |
| mass | | 0.13 | 0.18 | | -0.23 | 0.48 |
| range | | -0.01 | 0.07 | | -0.14 | 0.13 |
| migratory behaviour (resident) | | 0.17 | 0.53 | | -0.87 | 1.21 |
| migratory behaviour (resident):aspect ratio | | 0.14 | 0.47 | | -0.78 | 1.07 |
| migratory behaviour (resident):population | | -0.00 | 0.07 | | -0.13 | 0.13 |
| diet (herbivore) | | -0.11 | 0.38 | | -0.85 | 0.63 |
| diet (insectivore) | | -0.00 | 0.19 | | -0.37 | 0.37 |
| diet (omnivore) | | -0.05 | 0.20 | | -0.45 | 0.34 |
| foraging behaviour (tree foraging) | | -0.04 | 0.32 | | -0.66 | 0.58 |
| foraging behaviour (sallying) | | -0.03 | 0.18 | | -0.39 | 0.33 |
| foraging behaviour (aerial search) | | -0.02 | 0.15 | | -0.32 | 0.27 |
| foraging behaviour (aerial capture) | | -0.07 | 0.32 | | -0.70 | 0.57 |

Table S6.

Generalized variance inflation factors (GVIF) for variables in the aspect ratio model set. Last column allows for comparison of GVIFs between categorical and continuous variables.

| Variables | df | GVIF | (GVIF^1/(2*DF)^)^2^ |
| --- | --- | --- | --- |
| aspect ratio | 1 | 4.91 | 4.91 |
| population | 1 | 2.47 | 2.47 |
| habitat | 3 | 24.17 | 1.70 |
| foraging behaviour | 4 | 27.57 | 1.51 |
| mass | 1 | 2.15 | 2.15 |
| diet | 3 | 7.60 | 1.40 |
| migratory behaviour | 1 | 2.40 | 1.55 |
| range size | 1 | 1.52 | 1.52 |
| recovery year | 1 | 3.71 | 3.71 |

Table S7.

Single-predictor PGLS models of the relationship between natal dispersal distance and flight efficiency proxies. All flight efficiency proxies were natural log-transformed. Asterisks denote level of significance of a given coefficient; * = p < 0.05, ** = p < 0.005, *** = p < 0.0005. λ estimates the degree of phylogenetic non-independence in residuals on a scale from 0 to 1, Log(Lik) is the log-likelihood, ΔAICc is the difference between the AICc of the best model and the given model, *Pmodel* is the model probability, and *R^2^* is the coefficient of determination.

| Model | Intercept | Coefficient | df | λ | Log(Lik) | AICc | ΔAICc | *P_model_* | *R^2^* |
| --- | --- | --- | --- | --- | --- | --- | --- | --- | --- |
| aspect ratio | -0.73 | 2.12*** | 2 | 0.33 | -62.66 | 129.6 | 0 | 0.76 | 0.15 |
| lift-drag-ratio | -1.66 | 1.81*** | 2 | 0.29 | -63.83 | 132.5 | 2.34 | 0.27 | 0.11 |
| hand-wing index | -1.09 | 1.22* | 2 | 0.43 | -70.95 | 146.2 | 16.56 | <0.01 | 0.05 |
|  |  |  |  |  |  |  |  |  |  |

Table S8.

Coefficients for the single-predictor PGLS model of habitat predicting natal dispersal distances. Habitat category “coast” was set as the reference level.

|  | **β** | **Standard Error** | **t-value** | ***p*-value** |
| --- | --- | --- | --- | --- |
| intercept | 4.24 | 0.40 | 10.58 | <0.001 |
| habitat (open) | -0.81 | 0.49 | -1.76 | 0.08 |
| habitat (wetlands) | -0.76 | 0.40 | -1.91 | 0.06 |
| habitat (woodlands) | -1.94 | 0.49 | -3.98 | <0.001 |

Table S9.

Coefficients for the single-predictor PGLS model of foraging behaviour predicting natal dispersal distance. Foraging behaviour category “surface foraging” was set as the reference level.

|  | **β** | **Standard Error** | **t-value** | ***p*-value** |
| --- | --- | --- | --- | --- |
| intercept | 3.83 | 0.39 | 9.95 | <0.001 |
| foraging behaviour (tree foraging) | -1.20 | 1.08 | -1.11 | 0.27 |
| foraging behaviour (sallying) | -0.82 | 0.46 | -1.78 | 0.08 |
| foraging behaviour (aerial search) | 0.01 | 0.46 | 0.01 | 0.99 |
| foraging behaviour (aerial capture) | 0.99 | 0.54 | -1.83 | 0.07 |

Table S10.

Coefficients for the single-predictor PGLS model of diet predicting natal dispersal distance. Diet category “carnivore” was set as the reference level.

|  | **β** | **Standard Error** | **t-value** | ***p*-value** |
| --- | --- | --- | --- | --- |
| intercept | 3.85 | 0.39 | 9.87 | <0.001 |
| diet (herbivore) | -1.28 | 0.68 | -1.87 | 0.07 |
| diet (insectivore) | -0.43 | 0.47 | -0.92 | 0.36 |
| diet (omnivore) | -0.37 | 0.42 | -0.89 | 0.38 |

Table S11.

Coefficients for the single-predictor PGLS model of migratory behaviour predicting natal dispersal distance. Migration behaviour category “migrant” was set as the reference level.

|  | **β** | **Standard Error** | **t-value** | ***p*-value** |
| --- | --- | --- | --- | --- |
| intercept | 3.56 | 0.38 | 10.25 | <0.001 |
| migration behaviour (resident) | -0.37 | 0.47 | -0.79 | 0.44 |
